# Germination as a Bioprocess: Unraveling Its Impact on the Nutritional and Flavor Profile in Four Quinoa Beer Varieties

**DOI:** 10.3390/foods15081443

**Published:** 2026-04-21

**Authors:** Jiachen Xu, Yanling Zhang, Zhiyu Liu, Chaosheng Wu, Wei Wang, Xiao Feng, Caili Fu

**Affiliations:** 1 National University of Singapore (Suzhou) Research Institute, 377 Lin Quan Street, Suzhou Industrial Park, Suzhou 215123, China; 2 College of Food Science and Engineering/Collaborative Innovation Center for Modern Grain Circulation and Safety/Key Laboratory of Grains and Oils Quality Control and Processing, Nanjing University of Finance and Economics, No. 3 Wenyuan Road, Nanjing 210023, China; 3 Suzhou Institute for Food Control, No.6 XieXing Street, Xiangcheng District, Suzhou 215100, China

**Keywords:** quinoa beer, γ-aminobutyric acid, germination, anti-nutritional factors, phytic acid, flavor

## Abstract

Quinoa contains all the essential amino acids for human nutrition, which is also known to be gluten-free. In this research, black, red, white, and gray quinoa were germinated to ferment beers. The effects of germination as a bioprocess on the nutritional profile, anti-nutrients, and flavor development in quinoa beers were systematically investigated, and a comprehensive comparison was made with two commercially popular beers. The results indicated that the optimal germination time for quinoa in beer production was 48 h. Germination significantly increased the contents of polyphenols (255.9 mg/L in white quinoa beer) and flavonoids (404.34 mg/L in red quinoa beer), which enhanced the antioxidant activity of the beers. Furthermore, the levels of protein and γ-aminobutyric acid were elevated through germination. Notably, germination markedly improved the potential nutritional accessibility of the beers through reducing the anti-nutritional factors, including phytic acid, tannins, and trypsin inhibitor. In terms of flavor, quinoa beers developed a unique and pleasant aromatic profile, characterized by compounds such as ethyl octanoate, ethyl 9-decenoate, and ethyl pentadecanoate, which distinguished them from commercial beers. In conclusion, germinated quinoa can serve as a high-quality brewing material for producing beer with enhanced nutritional value, reduced anti-nutrients, and improved flavor characteristics.

## 1. Introduction

Being a pseudo-grain, quinoa belongs to the *Chenopodiaceae* family and is an ancient crop with perfect nutritional composition and antioxidant substances [1]. The Food and Agriculture Organization (FAO) has stated that quinoa is one of the ten globally healthy and nutritious foods that are most valuable for human consumption [2]. Quinoa seeds are the main edible parts of this plant, and there are generally four types of quinoa seeds, which are white, black, red, and gray quinoa. Quinoa has a protein content of 12–20%, and it contains all the essential amino acids. In addition, the long-term consumption of premium gluten-free quinoa contributes to the prevention of various diseases, including inflammation, obesity, diabetes, cancer, cardiovascular disease, and celiac disease [2].

Quinoa seeds typically contain over 50% starch (dry weight basis), which is predominantly amylopectin, making it a viable source of fermentable sugars [3]. Beer is the third most consumed beverage worldwide and contains various nutritionally relevant components, including soluble dietary fiber, essential minerals, and vitamins. Owing to the presence of these bioactive compounds, moderate beer consumption has been associated with potential health benefits, such as reduced risks of certain cancers and cardiovascular diseases, improved digestive function, and positive effects on kidney health. These properties are closely related to the brewing process, which influences the composition and bioavailability of health-related compounds in beer [3].

Quinoa is easy to germinate under mild conditions as a means of survival in high-stress habitats [4]. During germination, endogenous enzymes are activated, which leads to starch and protein hydrolysis. At the same time, the anti-nutritional factors such as phytic acid, tannins, and saponins decreased, while the content of γ-aminobutyric acid, vitamins, tocopherols, phenolic substances, and free amino acids increased [5,6]. Therefore, whether germinated quinoa can be an alternative to malted barley to generate beer products with improved nutritional values and decreased anti-nutritional factors is evaluated in this study.

There are limited studies on quinoa beers. Sun et al. [7] have investigated the innovation of quinoa beer, which used quinoa as a subsidiary, and studied the nutritional values and volatile compounds. Cela et al. [8] have investigated the gelatinized and ungelatinized form of quinoa beer, focusing on its physicochemical attributes and sensory properties. However, quinoa was not germinated as a raw material to ferment beers. The nutritional values and flavor of quinoa beers fermented solely from germinated quinoa remain unknown. The comparison with the commercial beers has not been reported.

In this study, quinoa beers are fermented from germinated white, black, red, and gray quinoa; no enzymatic supplementation was added during the brewing process. Different quinoa colors are known to vary in nutritional composition, which provides a broader perspective on how quinoa variety influences the nutritional and flavor profiles of the resulting beers. The distinct colors and associated flavor of quinoa may appeal to diverse consumer preferences, offering potential for product differentiation in the craft beer market. The nutritional values, anti-nutritional factors, and aroma of quinoa beers and two popular commercial beers are compared comprehensively. The effects of various germination times on the content of protein, free amino acids, polyphenols, and flavonoids in quinoa beers, as well as their antioxidant activity, are revealed. The effects of germination on the anti-nutritional factors in quinoa beers are also illustrated to optimize the germination conditions.

## 2. Materials and Methods

### 2.1. Materials

Quinoa was purchased from Beijing Jinhe Luyuan Trading Co., Ltd. (Beijing, China). Commercial beers were purchased from Qingdao Yikang Youpin Trading Co., Ltd. (Qingdao, China). All chemicals are of analytical grade.

### 2.2. Quinoa Germination

(1) Steeping:

Sodium hypochlorite solution of 10 g/L is used to sterilize the quinoa seeds for 10 min. The mass-to-volume ratio was 1:2.

(2) Germination:

After washing with deionized water, quinoa seeds were laid flat in a germination tray at 25 °C, and added with deionized water (1:2, *w*/*v*) for germination. The germination times of quinoa seeds were 0 h, 24 h, and 48 h, respectively. The seeds should be washed thoroughly every 24 h, with the water replenished to rinse them during the process. Germination time of 24 h and 48 h was selected based on preliminary studies and literature reports indicating that these intervals are sufficient to induce significant activation of endogenous enzymes (e.g., amylases, proteases, and phytase) and biochemical transformations in quinoa, while avoiding excessive rootlet growth or spoilage [8].

(3) Kilning:

After the predetermined germination time, the quinoa grains were removed and dried in a constant-temperature oven at 40 °C for 12 h.

### 2.3. Quinoa Beer Manufacture

In total, 50 g of germinated quinoa was broken into pieces by a crusher. The broken quinoa seeds were mixed with pure water (1:5, *w*/*w*), which were then placed in a water bath for saccharification to obtain wort. The quinoa wort was placed in a constant temperature water bath at 52 °C for 30 min, then heated at a rate of 1 °C per minute to 65 °C, held at 65 °C for 40 min, then further heated to 72 °C and held for 20 min, and finally heated to 78 °C and held for 10 min. The wort was filtered by filter paper before cooling until the filtrate was clarified. Then the wort was boiled, during which 0.15 g hop per 100 mL quinoa beer was added, and 0.1 g hop per 100 mL quinoa beer was added after boiling for 70 min. Cascade aromatic hops were selected. The mash was filtered through quantitative ashless filter paper (Whatman Grade 41, 20–25 µm pore size, Cytiva Bio-Tech Co., Ltd., Hangzhou, China). The wort was stirred and cooled to 23 °C. The Brix value of the wort was adjusted to 12 °Brix with white sugar. The active dry *Saccharomyces cerevisiae* yeast was added to sterile water at 35 °C, with the rehydration solution volume being five times the dry weight of the yeast, and the rehydration time was 30 min. After being rehydrated, it was added to the cooled sterile wort (0.6 g/L), and the wort was fermented at 20 °C for 14 days [9]. According to the method above, each 50 g of quinoa can be fermented to obtain approximately 100 mL of beer. From a fermentability perspective, according to Table 1 cited from Guardianelli, L.M. et al. [10], germination substantially increased the pool of readily fermentable carbohydrates. After 48 h of germination, the total monosaccharide content (glucose + fructose) increased by approximately 70–90%, while starch content decreased by up to 7.8% in red quinoa. This shift indicates a clear improvement in starch conversion efficiency, providing a more favorable carbohydrate profile for subsequent fermentation processes.

### 2.4. The Alcohol Content

The determination of alcohol content in beers is based on the density bottle method in GB/T 4928-2008, Methods of Beer Analysis [11].

### 2.5. The Total Phenolic and Flavonoid Content of Quinoa Beers

The total phenolic content in quinoa beer was determined using the Forinshawka method [12]. The total phenolic content was expressed as gallic acid equivalents (mg/L). The total flavonoid content was determined according to the method from Zlotek et al. [13] and was expressed as rutin equivalents (mg/L).

### 2.6. Antioxidant Activity of Quinoa Beers

The DPPH scavenging capacity was measured according to the method of Hirose, Fujita, Ishii, & Ueno [14].

### 2.7. γ-Aminobutyric Acid Content and Free Amino Acids Content of Quinoa Beers

The contents of γ-aminobutyric acid and free amino acids were determined using an automatic amino acid analyzer (Biochrom 30+, Biochrom, Cambridge, UK) [15]. In total, 1 mL of beer was diluted to 5 mL with ultra-pure water and mixed thoroughly by vortexing. From this dilution, 1 mL was taken and further diluted 10-fold with ultra-pure water. The resulting solution was filtered through a 0.22 μm membrane prior to injection into the analyzer.

### 2.8. Protein Content of Quinoa Beers

The protein content is determined using the Bradford method [16]. Each beer sample was appropriately diluted with phosphate-buffered saline (PBS). Then, 5 mL of the diluted Coomassie Brilliant Blue G-250 reagent (Shanghai Macklin Biochemical Co., Ltd., Shanghai, China) was added to the diluted sample, and the mixture was incubated at room temperature for 15 min. The absorbance was measured at 595 nm using a spectrophotometer. A standard curve was prepared using bovine serum albumin (BSA) to calculate protein concentration.

### 2.9. Anti-Nutritional Factors in Quinoa Beers

#### 2.9.1. Phytic Acid and Tannin Content

The total phytic acid in quinoa beers was determined according to the method from Wang & Guo [17]. The total tannin content in quinoa beer was determined using the Folin–Denis method [18].

#### 2.9.2. Trypsin Inhibitor Activity

The trypsin inhibitor activity content is determined using a newly developed dual-monoclonal sandwich ELISA method [19]. The Plant trasylol ELISA kit was used and purchased from Shanghai Mlbio(Shanghai Enzyme-linked Biotechnology Co., Ltd., Shanghai, China). The absorbance (OD) was measured at 450 nm using a microplate reader, and the concentration of the sample was calculated.

#### 2.9.3. Saponin Content

The perchloric acid colorimetric method was used for saponin content testing with appropriate modifications [20]. A homogenized quinoa beer sample of 1 mL was weighed and placed in a 50 mL volumetric flask. Five mL of water was added, followed by ultrasonic treatment for 30 min. The volume was then adjusted to 50 mL with water, which was mixed and filtered. A filtrate of 0.2 mL was taken and added to 0.2 mL of 5% vanillin-acetic acid solution. Subsequently, 0.8 mL of perchloric acid was added, and the mixture was homogenized and transferred to a 5 mL glass tube. The mixture was heated in a water bath at 60 °C for 10 min and then cooled in an ice bath. Glacial acetic acid of 5 mL was added and mixed well. The absorbance was measured at 560 nm.

Total saponin content was calculated according to the following equation:

X = *m*_1_ × V/(m × *V*_1_) × 100

In the equation,

X—Total saponin content in the sample, mg/100 mL;

m—Quality of the specimen, g;

$m_{1}$—Saponin mass of the specimen solution calculated on the standard curve, mg;

$V_{1}$—Volume of sample taken up for measurement, mL;

V—Extraction volume of the sample, mL.

### 2.10. Flavor Analysis of Quinoa Beers

Volatile components were quantified in duplicate by headspace solid-phase microextraction (HS-SPME) combined with gas chromatography (GC) and mass spectrometry (Agilent, Santa Clara, CA, USA) according to the literature [21].

### 2.11. Statistical Analysis

The results were expressed as mean value ± standard deviation. One-way analysis of variance (ANOVA) was used to determine the significant differences. Differences were considered significant at *p* < 0.05. Data analysis and processing were performed using SPSS 24 software (version 18.0). In this experiment, both the biological replicates (independent fermentations) and the analytical replicates were measured in triplicate, with the average value calculated from the results.

## 3. Results and Discussion

### 3.1. Effect of Different Germination Times on the Polyphenol and Flavonoid Concentration in Quinoa Beers

**Figure 1:**
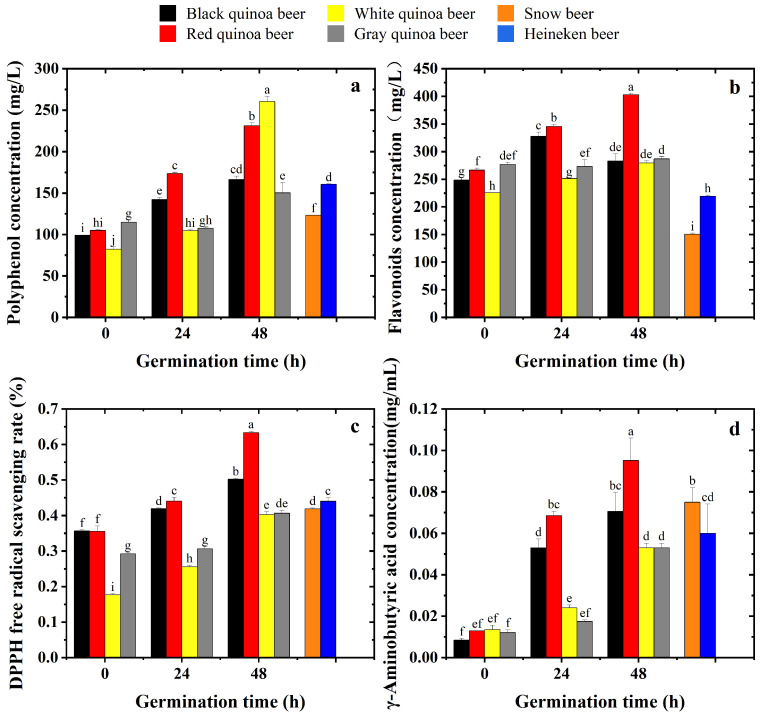
Effects of germination time on the biological values of quinoa beers. (**a**), Polyphenol content; (**b**), flavonoid content; (**c**), DPPH free radical scavenging rate; and (**d**), γ-aminobutyric acid content. Note: Values with superscript letters are significantly different (*p* < 0.05).

The nutritional values of beers fermented from quinoa germinated for 0 h, 24 h and 48 h are compared with those of commercial beers, as shown in Figure 1. In Figure 1a, red and white quinoa beers brewed from 48 h germinated quinoa showed significantly higher total phenolic content than the two commercial beers. The white quinoa beer brewed from 48 h germinated quinoa showed the highest total phenol content, but the 0 h white quinoa beer had the lowest total phenol content; this was due to the effects of germination. This is attributed to the activation of cellulase and β-glycosidase in quinoa, especially after 48 h of germination [5]. The activated cellulase can disrupt cell walls, releasing phenolic compounds that are attached to plant cell walls. Simultaneously, β-glycosidase aids in breaking β-glucosidic bonds, thus releasing phenolic compounds [22]. Therefore, the polyphenol content in quinoa beers increased with the extension of germination time. Additionally, the increase in polyphenol content may also result from the synthesis via the shikimate pathway during germination, utilizing specific amino acids and other phenolic acids as precursors [23]. The slight, non-significant decrease in TPC for gray quinoa beer at 24 h compared to 0 h may be attributed to sample heterogeneity or the leaching of some water-soluble phenolic compounds during the steeping and washing steps of germination, which could precede the subsequent biosynthesis and release of bound phenolics observed at 48 h [24].

As shown in Figure 1a, white quinoa beer brewed from 48 h germinated quinoa had the highest total polyphenol content, followed by red quinoa and black and gray quinoa beers brewed from 48 h germinated quinoa. Although red and black quinoa are richer in anthocyanin content compared to white quinoa, the higher phenolic acid content in white quinoa results in a greater total polyphenol content than in red and black quinoa [24]. Chen et al. [2] also reported that there were significant differences in phenolic compounds among different quinoa varieties, with values ranging from 34.52 ± 16.67 mg/100 g to 571.43 ± 14.29 mg/100 g based on the dry weight of quinoa. Moreover, Pacheco et al. [25] reported that germination increased the total phenolic content in different kinds of quinoa. Therefore, the variations in total polyphenol content in quinoa beers are related to the quinoa varieties and germination process.

Meanwhile, the fermentation process also increased the total phenolic content in quinoa beer, contributing to the production of health-promoting components such as γ-aminobutyric acid, flavonoids, and phenolic compounds. In non-fermented quinoa grains, phenolic compounds primarily exist in bound forms, conjugated with sugars, fatty acids, or proteins. The processes of glycosylation and conjugation influence the bioavailability and bioaccessibility of these compounds [26]. Through fermentation of quinoa, the bound forms of phenolic compounds undergo bioconversion into their free forms, enhancing their bioavailability and bioaccessibility in quinoa beers [27]. As shown in Figure 1b, the flavonoid content of each quinoa beer was significantly greater than that of two commercial beers. The flavonoid content of red quinoa beer was 404.340 mg/L and was significantly higher than that of the other three quinoa beers. Meanwhile, there was no significant difference in the flavonoid content of the black, white and gray quinoa beers. Figure 1b illustrates that germination can greatly enhance the flavonoid content of quinoa beers, especially for red quinoa. The increase in flavonoid content may be due to the catalytic effect of phenylalanine ammonia-lyase (PAL) and glycosylases [28]. In addition, during germination, the β-glycosidase is activated, which can aid in breaking β-glucosidic bonds and transform glycosides into flavonoid aglycones, leading to an increase in flavonoids in quinoa beer [29]. Compared with red quinoa beer, germination did not play a significant role in enhancing the flavonoid content in gray quinoa beer. The role of flavonoid subclasses is also worth noting. In quinoa beer, flavonoid subclasses such as catechins, epicatechins, rutin and quercetin have been identified, although many flavonoids occur at relatively low levels [30]. These flavonoid subclasses contribute differentially to antioxidant capacity, with compounds bearing multiple ortho-dihydroxy groups in the B-ring (e.g., catechin and quercetin) showing stronger correlations with in vitro antioxidant activity than total phenolic content alone [31].

### 3.2. Effect of Different Germination Times on the DPPH Free Radical Scavenging Rate of Quinoa Beers

Figure 1c shows the comparison of the antioxidant capacity between various quinoa beers, which is consistent with the results shown in Figure 1a,b. The extended germination time results in higher antioxidant capacity due to higher contents of flavonoids and polyphenols in quinoa beers. This demonstrated that germination offered a significant advantage over non-germination and that a germination duration of 48 h was a more favorable condition compared to 24 h.

As illustrated in Figure 1c, it is evident that the antioxidant activity of red and black quinoa beers brewed from quinoa germinated for 48 h was significantly higher compared to that of the two commercial beers. Meanwhile, red quinoa beer had significant higher DPPH scavenging rate over black quinoa beer. These results agree well with those presented by Tang et al. [32], who reported that white quinoa beer had relatively lower antioxidant activity, and gray quinoa beer showed the lowest antioxidant activity among quinoa beers. This proves that red and black quinoa is superior to barley to ferment beers in terms of antioxidant properties.

White quinoa beer had the highest phenolic content after 48 h germination, as shown in Figure 1a, while its antioxidant activity was relatively low. Red quinoa beer had the highest flavonoid content, as shown in Figure 1b, and it exhibited the highest antioxidant activity, which may be because flavonoids have a more significant contribution to the antioxidant capacity than polyphenols. However, this is inconsistent with the results of Zhou, Sun, Chen, & Li [33]. They reported that antioxidant capacity had the highest correlation with flavonoids and phenolics, and the coefficients were 0.886 and 0.973, respectively. It may depend on the present form of phenolic content in quinoa beer; quinoa beer phenolics are predominantly present as bound phenolic acids with limited intrinsic redox activity and reduced availability due to interactions with other matrix components [34]. It should be noted that assessing antioxidant capacity using a single method (DPPH) has its limitations. Future studies should employ multiple assays, such as ABTS and FRAP, for a more comprehensive evaluation.

### 3.3. Effect of Different Germination Times on the γ-Aminobutyric Acid Concentration

γ-Aminobutyric acid (GABA) is a naturally occurring nonprotein organic amino acid that acts as an inhibitory neurotransmitter in the mammalian central nervous system [2]. It has many functional properties to benefit human health, such as regulating heart rate and blood pressure and relieving anxiety and pain. Therefore, a number of studies have been carried out to enrich plant protein foods with GABA. As shown in Figure 1d, the difference in the content of GABA between the four quinoa beers was not significant when the germination time was 0 h. It is noteworthy that the GABA content of four quinoa beers gradually increased with the extended germination time. It was reported that germination of quinoa seeds activates glutamate decarboxylase (GAD), GABA transaminase (GABA-T), and succinate-semialdehyde dehydrogenase (SSADH), leading to the conversion of glutamic acid into GABA. This enzymatic process may be regulated by glutamate decarboxylase 8 (CqGAD8) and GABA transaminase 2 (CqGABA-T2) genes [23]. Overall, germination for 48 h was the optimal condition for quinoa beer production in terms of GABA content, and red quinoa beer showed significantly higher GABA content compared with two commercial beers.

The GABA content of red quinoa beer fermented from 48 h germinated quinoa was 6.69 times greater than that of red quinoa beer brewed from ungerminated quinoa. This result is similar to the results of Zhang et al. [15], who found that the GABA content in germinated quinoa was nearly twice as high as that of the raw quinoa seeds. The red quinoa beer had the highest content of GABA of 0.0874 mg/mL and 0.0710 mg/mL when the germination time was 48 h and 24 h. Meanwhile, the GABA content in black quinoa beer was slightly lower than that of red quinoa beer, but was significantly higher than that of white and gray quinoa beers after 24 h and 48 h germination. The difference in GABA content of quinoa is caused by various expressions of quinoa genes, including eight GAD genes, two GABA-T genes, one SSADH gene, nine polyamine oxidase (PAO) genes, and five diamine oxidase (DAO) genes [15].

### 3.4. Effects of Different Germination Times on the Protein and Amino Acid Contents of Quinoa Beers

**Figure 2:**
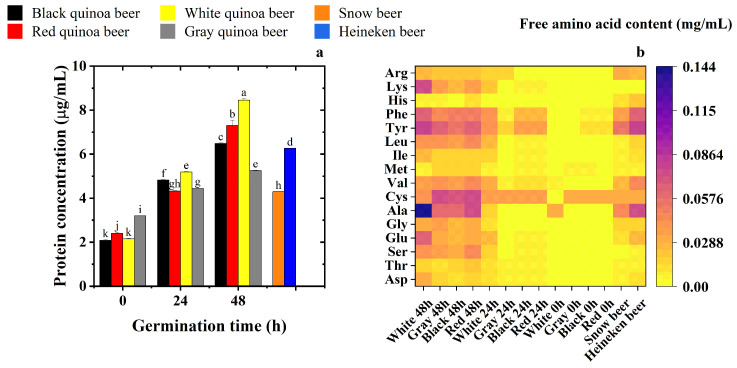
Effects of germination on the protein content and free amino acid content of quinoa beers. (**a**), Protein content and (**b**), free amino acid content. Note: Values with superscript letters are significantly different (*p* < 0.05).

As Figure 2a shows, germination can substantially increase the protein content in four types of quinoa beers, especially white quinoa beer. The protein content of white quinoa beer brewed after 48 h of germination was 8.505 µg/mL (equivalent to 0.85 mg/100 mL), approximately four times higher than that of quinoa beer fermented from non-germinated white quinoa and one-quarter of the protein content in milk (approximately 3.2 mg/100 mL) [35]. The protein content of red quinoa and black quinoa beer was lower than that of white quinoa beer, while gray quinoa had the lowest protein content after 48 h of germination. White, red, and black quinoa beers brewed after 48 h germination showed significantly higher protein content than the two commercial beers. The increase in protein content in quinoa beers during germination time was due to the endogenous enzymes being activated, synthesizing new proteins and mobilizing reserve proteins located in the cotyledons of the quinoa grains [23]. Meanwhile, the nitrogen stored in the quinoa seeds was mobilized, which allowed a significant increase in protein [36].

The result of protein content in quinoa beers is consistent with Figure 2b, where white quinoa beer brewed after 48 h germination showed the highest free amino acid content, followed by red and black quinoa beers. Free amino acids play a crucial role in seed germination [37]. According to Figure 2b, beers brewed from ungerminated quinoa or 24 h germinated quinoa were generally lower in free amino acids. Lan et al. [23] found that germination activates proteases, resulting in the production of peptides and free amino acids. Compared to commercial Snow and Heineken beers, beers fermented from 48 h germinated quinoa showed a small advantage in terms of free amino acids composition. Two commercial beers lacked lysine, which is a limiting amino acid in most cereals. Lysine is one of the eight essential amino acids; it is not synthesized by the body [38]. However, all quinoa beers contained lysine, and the concentration was 0.131 mg/mL, 0.025 mg/mL, 0.036 mg/mL, and 0.035 mg/mL for white, black, red and gray quinoa beers. The balanced amino acid composition of quinoa seeds significantly improved the nutritional values of quinoa beers, as quinoa beers contained all kinds of essential amino acids.

Furthermore, Snow beer lacks serine, with a concentration of 0.004 mg/mL. However, four types of quinoa beers brewed after 48 h showed significantly higher content of serine from 0.006 to 0.043 mg/mL. Serine plays an important role in cellular functions, including protein synthesis, neurotransmission, and folate and methionine cycles, as well as the synthesis of sphingolipids, phospholipids, and sulphur-containing amino acids [39]. Moreover, it is noteworthy that Snow and Heineken beers both showed low concentrations of Cysteine, which were 0.003 and 0.004 mg/mL, respectively, and were far lower than the four types of quinoa beers brewed after 48 h, ranging from 0.033 mg/mL to 0.074 mg/mL. Meanwhile, four types of quinoa beers brewed after 48 h showed significantly higher content of Isoleucine from 0.016 mg/mL to 0.025 mg/mL compared with Snow beer of 0.001 mg/mL and Heineken beer of 0.007 mg/mL. Isoleucine was also a branched-chain amino acid, as well as leucine (Leu) and valine (Val), and it can undergo metabolism by yeasts to yield specific aldehydes during the beer storage process, which impart malty, fruity, and buttery aromas [40]. Moreover, quinoa beers were rich in aromatic amino acids, such as phenylalanine (Phe) and tyrosine (Tyr), which may be metabolized by yeasts into compounds that contribute to rosy and floral flavor [41]. All in all, quinoa beers fermented after 48 h of germination showed high and balanced free amino acids concentration, including all kinds of essential amino acids for human health.

### 3.5. Effects of Germination on the Anti-Nutritional Factors of Quinoa Beers

#### 3.5.1. Effect of Different Germination Times on the Phytic Acid in Quinoa Beers

**Figure 3:**
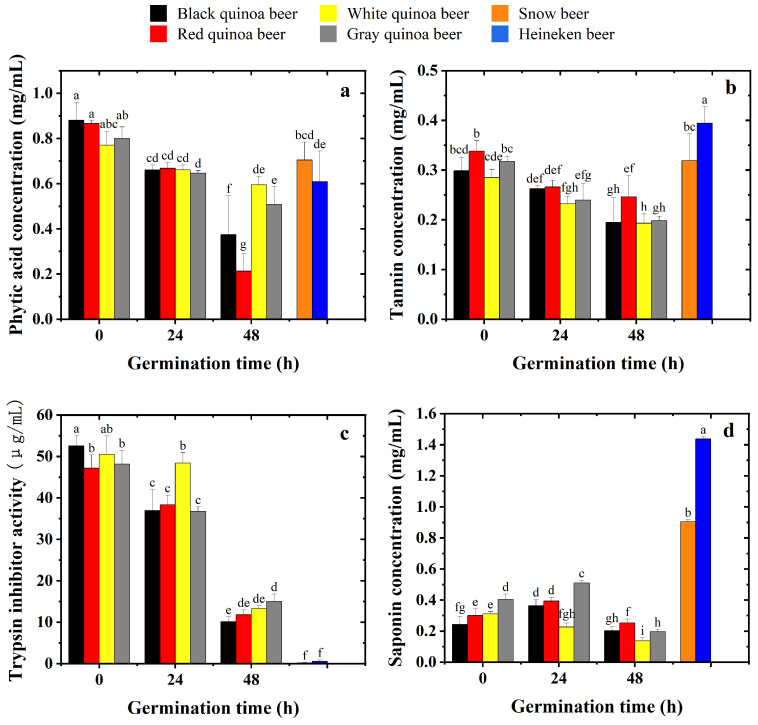
Effects of germination on the anti-nutritional factors of quinoa beers. (**a**), Phytic acid content; (**b**), tannin content; (**c**), trypsin inhibitor activity; and (**d**), saponin content. Note: Values with superscript letters are significantly different (*p* < 0.05).

The anti-nutritional factors in beers fermented from quinoa germinated for 0 h, 24 h and 48 h, and two commercial beers are shown in Figure 3. Phytic acid is a saturated cyclic acid with six active phosphate groups and binds positively charged functional groups or minerals as a chelating agent, which limits the bioavailability of minerals [42]. In Figure 3a, black, red and gray quinoa beers all showed a significant decrease in phytic acid concentration with the increase in germination time. Meanwhile, black quinoa beer showed a significant decrease in phytic acid concentration after 24 h of germination. These results indicated that the germination process enhanced the phytase activity within the seeds, which effectively degraded phytic acid [43]. The most significant decrease in phytic acid concentration was observed in red quinoa beer after 48 h of germination, from 0.866 ± 0.007 mg/mL to 0.213 ± 0.044 mg/mL. This may be due to the higher phytase activity in the red quinoa seeds [44]. The loss of phytic acid in QPI gels was related to the increase in phytase activity in germinated quinoa to hydrolyze phytic acid, leading to the increased mineral bioavailability [45]. During germination, endogenous enzymes are activated, which hydrolyze and degrade anti-nutritional factors. Specifically, phytase breaks down phytic acid, while other enzymes, such as polyphenol oxidase or tannase, reduce tannin content, and glycosidases or esterases cleave saponins. This enzymatic degradation lowers the levels of these compounds, thereby reducing their mineral-chelating effects and enhancing the bioavailability of minerals like Fe, Ca, and Zn [5].

It is noteworthy that black and red quinoa beers fermented from 48 h germinated quinoa had significantly lower phytic acid concentrations compared with two commercial beers. This indicates that germination effectively reduced the phytic acid concentration of black and red quinoa beers, thus eliminating the issue of mineral chelation. According to Panda et al., the dietary intake of omnivores consumes 150–1400 mg/day, compared with the quinoa beers after 48 h germination, which were below 0.6 mg/mL. The values remained far below this criterion [46].

#### 3.5.2. Effect of Different Germination Times on the Tannin Acid Concentration in Quinoa Beers

Tannin concentration in different quinoa beers and two commercial beers was measured, and the results are shown in Figure 3b. Tannins are polyphenols that form complexes with proteins, thereby interfering with their digestion and absorption [42]. Interference with the digestion and absorption of proteins may lead to indigestion. Overall, the tannin concentrations of all four quinoa beers decreased significantly with the extension of germination time. Red and white quinoa beers fermented from 24 h germinated quinoa had significantly lower tannin concentrations than quinoa beers fermented from quinoa without germination. After 48 h of germination, the tannin concentration of gray quinoa beer decreased from 0.318 ± 0.006 mg/mL to 0.198 ± 0.005 mg/mL. Similar to previous findings [47], seeds soaked in water and germinated were effective in reducing tannin content. The decrease in tannin content observed after germination was attributed to the formation of tannin–protein complexes in the plant matrix [48]. It is noteworthy that the tannic acid concentration in four quinoa beers is lower than in two commercial beers. Germination for 48 h further lowered the tannic acid content. Tannins give foods an astringent flavor and impair the absorption of vitamin B_12_, iron and glucose [49]. Germination effectively reduced the tannin concentration in the fermented quinoa beers, improving their taste and reducing the anti-nutritional factors. Meanwhile, Ahmed et al. showed that a safe tannin consumption level of 1.5–2.5 g/day is generally considered acceptable for the general population. Since the quinoa beer after 48 h germination was around 0.2 mg/mL, which was well below this standard, with reasonable consumption of quinoa beers.

#### 3.5.3. Effect of Different Germination Times on the Trypsin Inhibitor Activity of Quinoa Beers

Trypsin inhibitor activity of quinoa beers was measured, and the results are shown in Figure 3c. Quinoa germination for 24 h and 48 h significantly decreased the trypsin inhibitor activity of quinoa beers. Among them, black quinoa beer showed the most significant decrease in trypsin inhibitor activity after 48 h of germination, from 52.592 ± 1.418 μg/mL (0 h germination) to 10.151 ± 0.656 μg/mL. Trypsin inhibitors can be involved in complex physiological metabolism during the early stages of germination, leading to a reduction in trypsin inhibitor content [50]. However, there was no significant decrease in trypsin inhibitor activity of white quinoa beers fermented from 24 h germinated quinoa compared with beers brewed from non-germinated quinoa. Moreover, white quinoa beer was less effective at degrading trypsin inhibitors during 0–24 h germination. However, germination for 48 h effectively degraded the trypsin inhibitors in all four kinds of quinoa beers. Therefore, germination for 48 h can significantly improve the in vitro digestibility and decrease the antinutritional factor in quinoa beers.

#### 3.5.4. Effect of Different Germination Times on the Saponin Concentration in Quinoa Beers

In Figure 3d, the saponin content of black, red and gray quinoa beers increased firstly and then decreased with the increasing germination time of quinoa. Similar to previous findings [51], the saponin content of soybeans increased after germination. The increase in saponin content during quinoa germination may be due to the synthesis and activation of different enzyme systems that promote saponin production. It is also possible that the weakening of the seed structure enhances the solvent extraction process [52]. The saponin content of white quinoa beer decreased from 0.310 ± 0.010 mg/mL to 0.137 ± 0.012 mg/mL after 48 h of germination. There may be differences in the biosynthesis and regulation of saponins in different quinoa varieties, which may explain the continued decline in the saponin content of white quinoa beer. The bitterness threshold for saponins in quinoa is 1.1 mg/mL [52], and gastrointestinal effects in vitro occur above approximately 0.86 mg/mL [53]. The saponin content in our germinated white quinoa beer was 0.137 mg/mL, and even the highest level observed was less than 0.5 mg/mL, which remains well below both thresholds. Thus, germination yields a functional beverage with saponin levels that are safe for consumption and are sensory-acceptable. The saponin concentrations of the four quinoa beers brewed after 48 h of germination were significantly lower than those of the two commercial beers. The reduction in saponin concentration reduced the bitter flavor in the quinoa beers and showed a significant advantage over Snow and Heineken beers. Saponins were widely found in cereals, such as sorghum and barley, which were raw ingredients used to brew beers [54,55]. Saponins can be removed by a variety of methods, including soaking, grinding, germination, and polishing [56]. During polishing, the outer layer of quinoa seeds was removed by rubbing, leading to the 88.49% removal of saponin in the quinoa grains. Therefore, a lower saponin content was observed in quinoa beers compared with commercial beers [57].

### 3.6. Alcohol Content of Quinoa Beers and Commercial Beers

**Figure 4:**
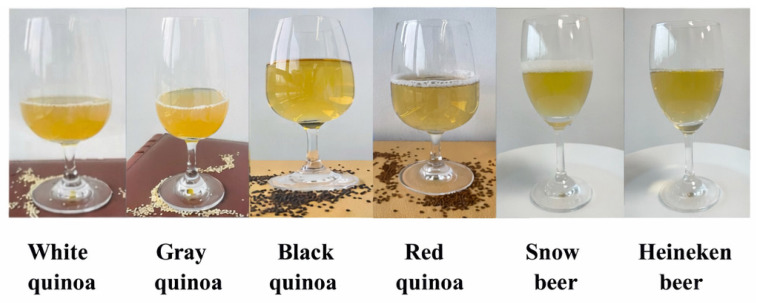
The appearance of beers fermented from germinated quinoa seeds.

**Figure 5:**
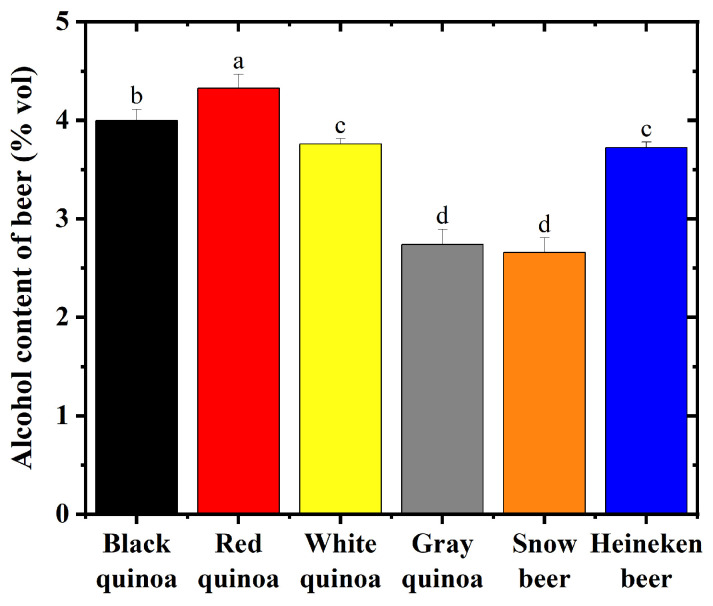
Alcohol concentration of beers fermented from quinoa germinated for 48 h. Note: Values with superscript letters are significantly different (*p* < 0.05).

According to the results and discussions above, quinoa beers fermented from 48 h germinated quinoa exhibited increased polyphenol, flavonoid, GABA, and protein content, along with reduced phytic acid, tannin content, and trypsin inhibitor activity. Therefore, we compared the appearance and alcohol content of four quinoa beers with the optimal germination time to those of two commercial beers. The appearance of beers fermented from germinated quinoa seeds is shown in Figure 4. The appearance of quinoa beers is appealing and similar to that of commercial beers. After the primary fermentation (14 days), the beer was decanted from the yeast sediment, and no further filtration or clarification agents were used. The clarity observed in Figure 4 is attributed to this careful decanting and the flocculation characteristics of the US-05 yeast strain. White and gray quinoa beers showed higher yellowness compared with commercial beers, while black and red quinoa beers had higher transparency than commercial beers. However, commercial beers showed better foaming properties. As shown in Figure 5, after 14 days of fermentation, white, black, and red quinoa beers had higher alcohol content than Snow beer in the market. White quinoa beer showed comparable alcohol content with Heineken beer (3.78% vol). In contrast, gray quinoa beer exhibited a significantly lower alcohol content (2.74% vol) compared to the other quinoa beers. The yeast in gray quinoa beer was found to utilize sugar efficiently, suggesting that fermentation was primarily characterized by an extended aerobic phase and a relatively shorter anaerobic phase, which may contribute to its lower alcohol production [58].

### 3.7. Aroma Compound Analysis of Quinoa Beers and Two Commercial Beers

There are aroma compounds contained in all six beers, such as nonanal, decanal, 1-butanol, 3-methyl-, acetate, hexanoic acid, ethyl ester, acetic acid, 2-phenylethyl ester, hexadecanoic acid, ethyl ester, octadecanoic acid, ethyl ester and 2,4-di-tert-butylphenol, among which 1-butanol, 3-methyl-, acetate have a sweet fruity and ripe banana aroma. Acetic acid 2-phenylethyl ester has a floral aroma similar to that of roses, with sweet honey notes [59]. Decanal has a fresh orange and orange peel aroma. Nonanal has a rose and citrusy aroma with a strong oily note, and is also found in black and green tea, giving a specific sensory experience.

The formation of aroma compounds is complex, and beer aroma substances are very important because they contribute significantly to the quality of the final product. More than 1000 compounds have been found in beer, either from raw materials such as hops or from products formed during processing, especially during fermentation [60]. The results of GC–MS for black, red, white and gray quinoa beers, as well as for Snow beer and Heineken beers, are shown in Table 2. After comparing the aroma compounds in different beers using the OPLS-DA model, it shows that 2,6-nonadienal, 1-pentanol, phenyl ethanol, isoamyl acetate, ethyl caprate, butyl butyrate, ethyl propionate, furfuryl alcohol, phenethyl acetate, ethyl laurate, ethyl butyrate, acetic acid, and 3-methyl-4 heptanone are the key aroma components that can distinguish among different types of beers [61]. These compounds contribute to the diversity of aroma, influencing the consumer’s overall sensory experience. Aroma was distinguished by comparing the similarities and differences in aroma compounds, especially some of the substances included in key aroma compounds.

It is noteworthy that there are a number of aroma compounds that only four types of quinoa beers contain, while Snow beers and Heineken beers do not, such as ethyl 9-decenoate and octanoic acid, ethyl ester. Ethyl 9-decenoate has a fruity aroma similar to that of pear and apple, which provides quinoa beers with an appealing fruity odor [62]. Octanoic acid, ethyl ester has a fruity aroma when diluted. Therefore, quinoa beers possessed more abundant and pleasant aroma compounds compared with two popular commercial beers, making it has great potential in the food market.

There are also aroma compounds found only in certain beers. For example, 3-methyl-1-butanol, which imparts banana, liqueur, and malty aromas, is present in all beers except Heineken beer. Black, red and gray quinoa beers and Snow beer all contain 1-octanol, which has a citrusy aroma when diluted. Red and gray quinoa beers, Snow beer, and Heineken beer all contain 3,4-dimethylbenzaldehyde, which has a sweet almond aroma. With the exception of black quinoa beer, other beers contain α-methyl-benzenemethanol, which has a floral aroma. Meanwhile, except for red quinoa beer, other beers contain ethyl acetate, which has a fruity aroma. Red and gray quinoa beers, Snow beer, and Heineken beer contain humulene epoxide i, which has a herbal aroma of hops [63]. All beers except Snow beer contain phenylethyl alcohol, which has a sweet, rose-like floral aroma. To conclude, quinoa beers have a more complex and diverse aroma profile than the two commercial beers. Meanwhile, there are specific compounds in quinoa beers that bring out unique aromas that distinguish them from commercial beers, such as fruity aroma and rose aroma, making them more pleasing and unique to consumers.

## 4. Conclusions

Germination significantly improved the quality of quinoa beers, which contained all kinds of essential amino acids. Meanwhile, quinoa beers fermented from 48 h germinated quinoa showed an advantage over beers brewed from 24 h germinated quinoa.

Technologically, the process efficiently liberated and increased key bioactive compounds without exogenous enzymatic supplementation. Notably, varietal differences were evident, with white quinoa yielding the highest protein and amino acid enrichment, while red quinoa provided superior flavonoid content and antioxidant activity.

Concurrently, germination significantly reduced anti-nutritional factors such as phytic acid, tannins, and trypsin inhibitors. This reduction mitigates potential mineral chelation and protein digestibility issues, thereby improving the nutritional accessibility of the final product.

Aroma compound analysis further confirmed the value of this approach. The unique volatile compound profile, characterized by esters like ethyl octanoate and ethyl 9-decenoate, imparted a distinct and pleasant aroma, differentiating quinoa beers from their commercial counterparts and suggesting strong potential for consumer appeal.

To sum up, germinated white, black, red, and gray quinoa can be an alternative to malted barley to ferment beers with enhanced nutritional values, decreased antinutritional values, and improved aroma.

## Figures and Tables

**Table 1:** Available carbohydrate content of ungerminated and germinated quinoa [10].

Flours	Fructose (%)	Glucose (%)	Sucrose (%)	Starch (%)
WQ	0.22 ± 0.04 a	0.91 ± 0.01 a	0.80 ± 0.10 a	57.72 ± 0.78 a
WQ 18	0.34 ± 0.03 b	1.49 ± 0.01 b	0.92 ± 0.06 ab	57.72 ± 0.82 a
WQ 24	0.44 ± 0.02 c	1.53 ± 0.02 bc	0.96 ± 0.12 ab	59.63 ± 0.53 b
WQ 48	0.53 ± 0.02 d	1.58 ± 0.01 c	1.00 ± 0.08 b	57.06 ± 0.84 a
RQ	0.18 ± 0.01 a	0.80 ± 0.00 a	0.61 ± 0.10 a	53.11 ± 1.71 b
RQ 18	0.30 ± 0.01 b	1.46 ± 0.01 b	0.89 ± 0.10 b	56.2 ± 1.5 c
RQ 24	0.37 ± 0.01 c	1.51 ± 0.07 b	0.98 ± 0.05 b	54.93 ± 0.91 b
RQ 48	0.48 ± 0.01 d	1.52 ± 0.01 b	0.96 ± 0.07 b	48.97 ± 0.98 a

WQ 18, WQ 24 and WQ 48 for white quinoa germinated for 18, 24, and 48 h, respectively; RQ 18, RQ 24, and RQ 48 correspond to red quinoa germinated for 18, 24, and 48 h, respectively. Lowercase letters indicate significant differences among groups within the same column.

**Table 2:** Aroma compound analysis of quinoa beers and two commercial beers.

				Percentage (%)					
	No.	Name	Odor Description	Black Quinoa Beer	Red Quinoa Beer	White Quinoa Beer	Gray Quinoa Beer	Snow Beer	Heineken Beer
Alcohols	1	1-pentanol	mild fermented aroma	3.68 ^a^	5.00 ^a^	/	/	4.05 ^a^	3.24 ^a^
	2	3-methyl-1-butanol	banana, liqueur and malty aroma	17.60 ^a^	12.24 ^c^	14.66 ^b^	4.95 ^e^	9.16 ^d^	/
	3	α-methyl-benzenemethanol	floral, woody, sweet and warm	/	0.39 ^b^	1.44 ^b^	0.16 ^b^	21.98 ^a^	0.24 ^b^
	4	1-octanol	citrusy aroma	0.06 ^a^	0.07 ^a^	/	0.09 ^a^	0.08 ^a^	/
	5	phenylethyl alcohol	rose fragrance and sweetness	9.40 ^c^	8.00 ^c^	30.23 ^b^	36.69 ^a^	/	34.84 ^a^
Aldehyde	1	nonanal	citrus aroma	0.13 ^a^	0.13 ^a^	0.15 ^a^	0.15 ^a^	0.25 ^a^	0.20 ^a^
	2	decanal	fresh orange and orange peel aroma	0.06 ^b^	0.05 ^b^	0.06 ^b^	0.05 ^b^	0.15 ^a^	0.12 ^ab^
	3	3,4-dimethylbenzaldehyde	sweet almond aroma	/	10.15 ^c^	/	0.36 ^d^	23.12 ^a^	21.35 ^b^
	4	pentadecanal	soft and fruity	0.02 ^a^	0.15 ^a^	0.06 ^a^	0.18 ^a^	/	/
	5	3,5-di-tert-butyl-4-hydroxybenzaldehyde	stable aromatic aroma	0.22 ^a^	0.26 ^a^	0.18 ^a^	0.18 ^a^	/	/
Alkenes	1	(1r,3e,7e,11r)-1,5,5,8-tetramethyl-12-oxabicyclo [9.1.0]dodeca-3,7-diene	herbaceous and woody aroma	0.98 ^a^	1.00 ^a^	1.05 ^a^	2.75 ^a^	/	/
	2	humulene epoxide i	the herbal aroma of hops	/	0.36 ^a^	/	0.30 ^ab^	0.10 ^b^	0.26 ^ab^
	3	humulenol-ii	woody notes combined with herbal notes	7.25 ^a^	7.52 ^a^	6.12 ^ab^	4.96 ^b^	/	/
	4	14-hydroxycaryophyllene	complex spice aroma	0.19 ^a^	0.36 ^a^	0.17 ^a^	0.30 ^a^	/	/
Esters	1	ethyl acetate	fruity aroma	0.44 ^b^	/	0.32 ^bc^	0.22 ^c^	0.77 ^a^	0.31 ^bc^
	2	1-butanol, 3-methyl-, acetate	sweet fruity notes of a ripe banana	0.26 ^c^	0.09 ^c^	0.44 ^c^	0.33 ^c^	5.68 ^a^	3.21 ^b^
	3	hexanoic acid, ethyl ester	sweet fruity notes of green apple and pineapple	0.25 ^bc^	0.11 ^cd^	0.44 ^a^	0.27 ^b^	0.12 ^cd^	0.07 ^d^
	4	octanoic acid, ethyl ester	tropical fruit aromas such as pineapple or mango	1.88 ^a^	1.10 ^a^	1.88 ^a^	1.96 ^a^	/	/
	5	acetic acid, 2-phenylethyl ester	sweet honey and rose scent	6.58 ^e^	9.18 ^d^	11.93 ^c^	7.51 ^de^	28.30 ^a^	26.12 ^b^
	6	ethyl 9-decenoate	sweet pear and apple aromas	0.18 ^b^	0.21 ^b^	0.20 ^b^	0.43 ^a^	/	/
	7	dodecanoic acid, ethyl ester	coconut and creamy aroma, slightly fatty	0.55 ^b^	3.76 ^a^	3.87 ^a^	4.00 ^a^	/	/
	8	tetradecanoic acid, ethyl ester	mild creamy aroma with fatty notes	1.17 ^c^	4.06 ^b^	1.22 ^c^	5.01 ^a^	/	0.04 ^d^
	9	1,2-benzenedicarboxylic acid, bis(2-methylpropyl) ester	floral aroma with sweetness	/	0.26 ^a^	0.22 ^a^	/	0.19 ^a^	0.18 ^a^
	10	pentadecanoic acid, ethyl ester	creamy with faint fruity notes	0.17 ^b^	0.35 ^b^	0.24 ^b^	0.69 ^a^	/	/
	11	ethyl 9-hexadecenoate	creamy and floral notes	4.87 ^a^	5.30 ^a^	3.93 ^a^	4.44 ^a^	/	/
	12	hexadecanoic acid, ethyl ester	waxy and creamy aroma	23.50 ^a^	22.50 ^a^	13.58 ^c^	17.20 ^b^	1.20 ^e^	4.39 ^d^
	13	octadecanoic acid, ethyl ester	soft fatty acid aroma and sweet	17.64 ^a^	2.87 ^bc^	2.97 ^b^	2.22 ^bcd^	0.24 ^d^	0.92 ^cd^
Ketone	1	3,5-di-tert-butyl-4-hydroxyacetophenone	soft vanilla aroma	0.22 ^a^	0.26 ^a^	0.18 ^a^	0.18 ^a^	/	/
Phenol	1	2,4-di-tert-butylphenol	woodsy scent	2.69 ^a^	4.28 ^a^	4.48 ^a^	4.42 ^a^	4.61 ^a^	4.51 ^a^

Note: Values with superscript letters are significantly different (*p* < 0.05).

## Data Availability

The original contributions presented in this study are included in the article. Further inquiries can be directed to the corresponding authors.
